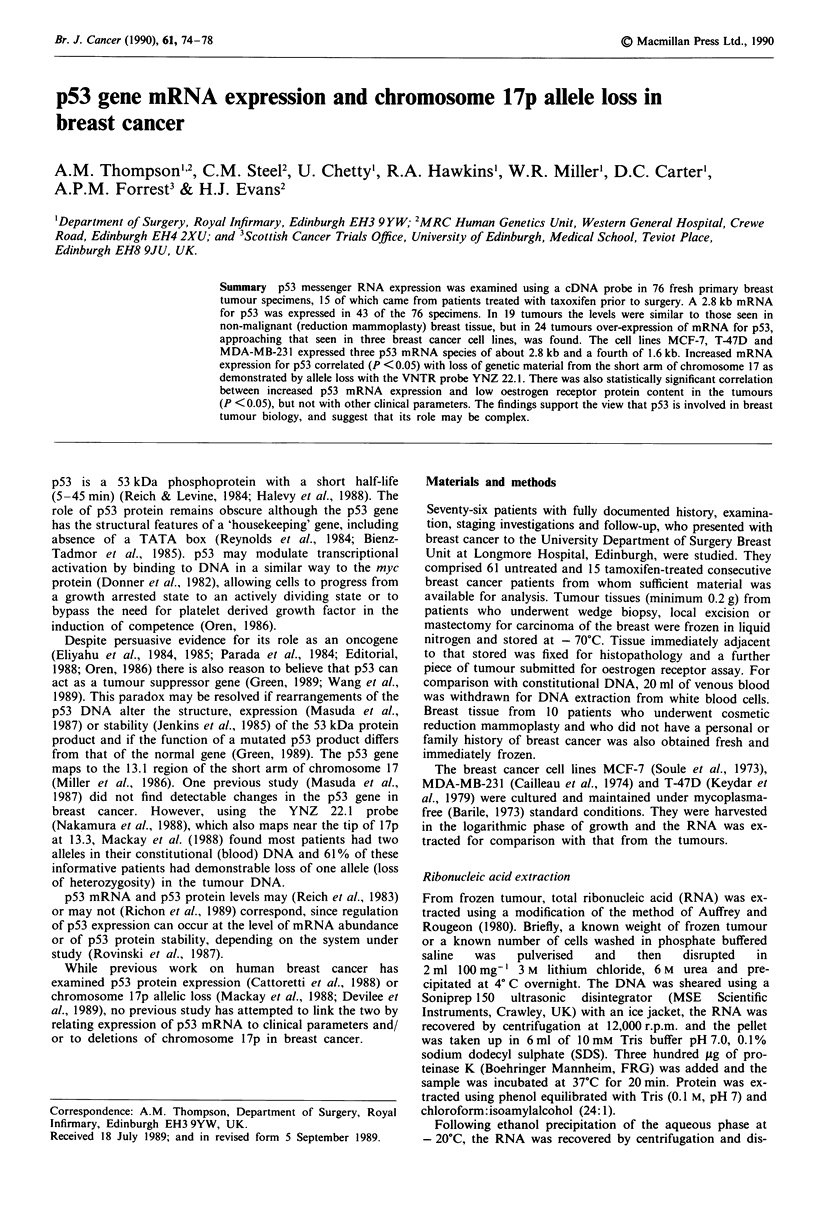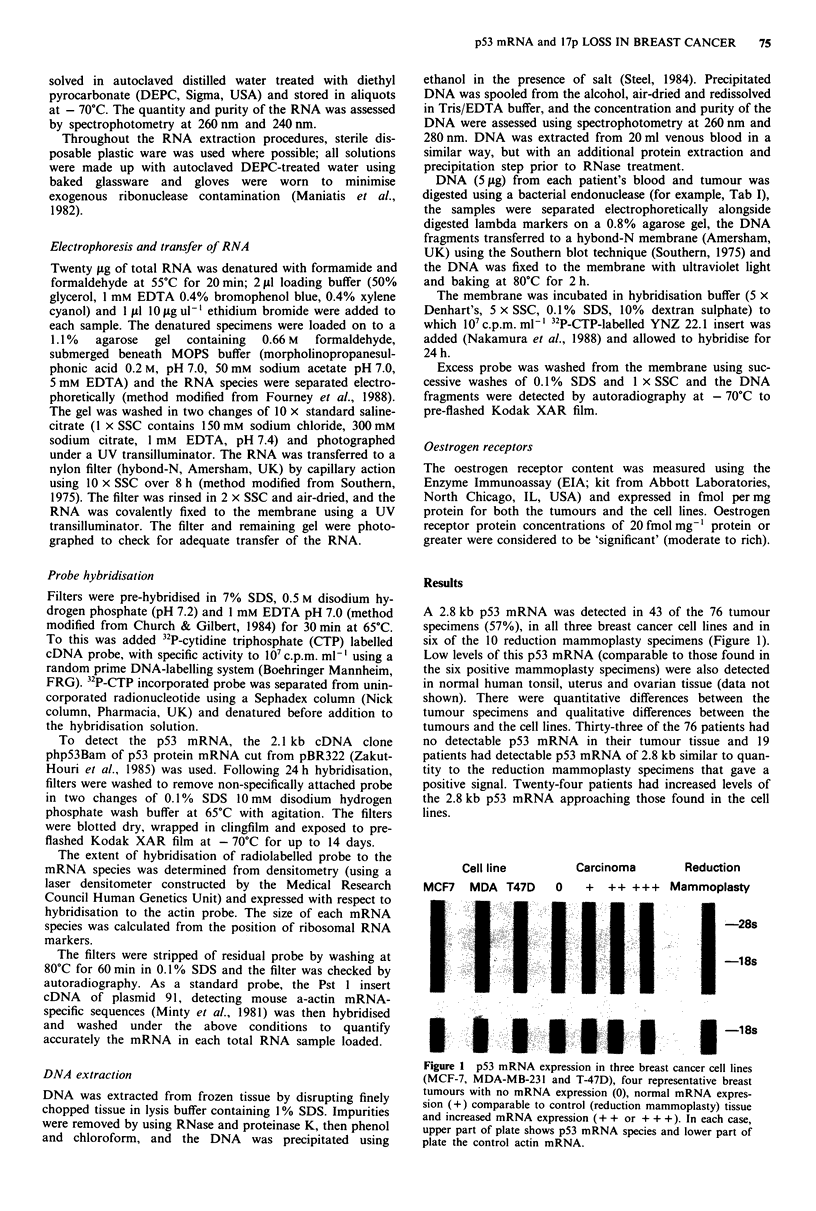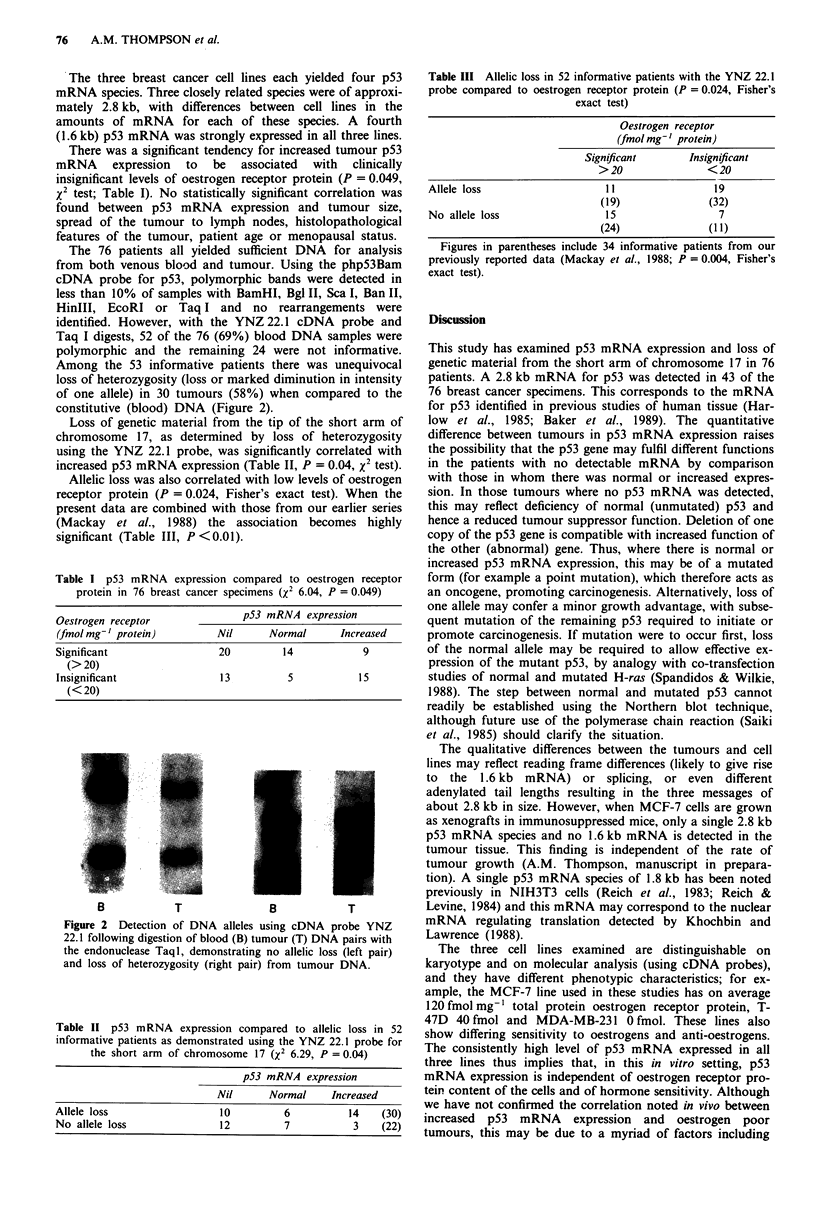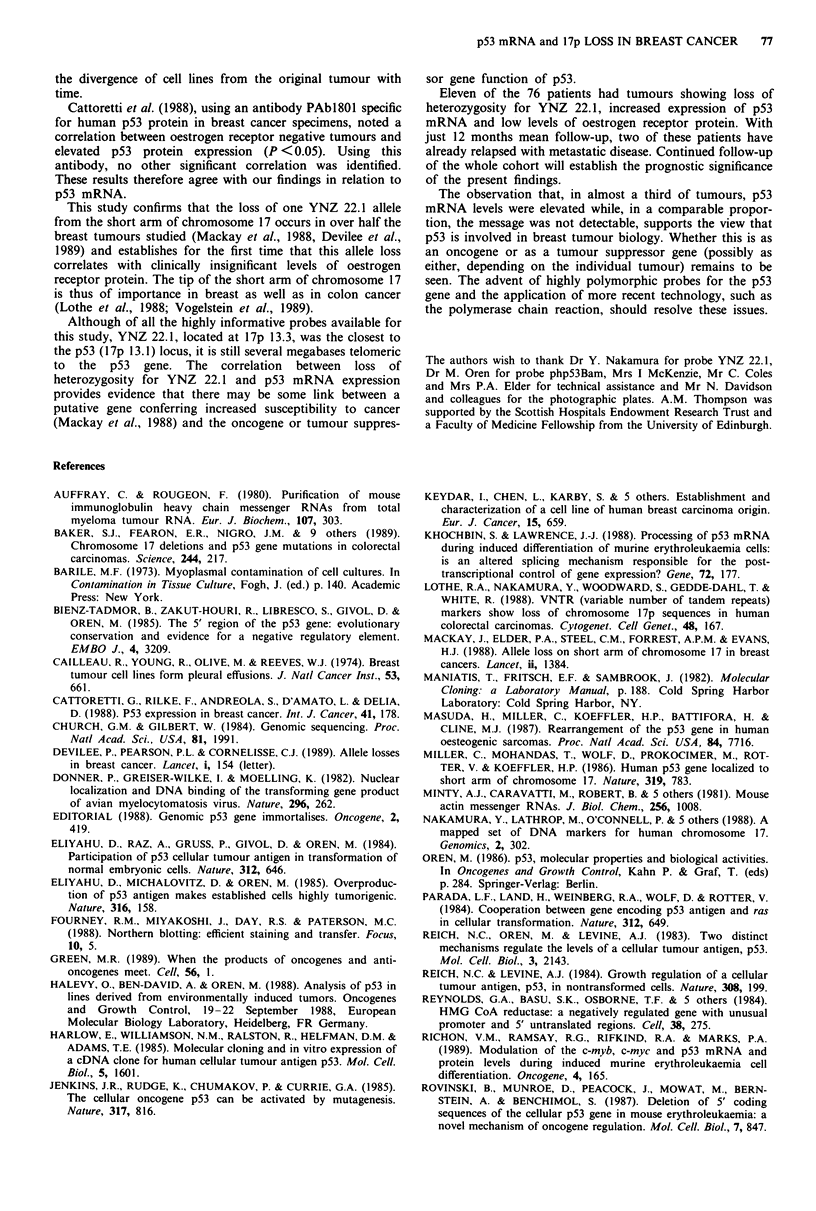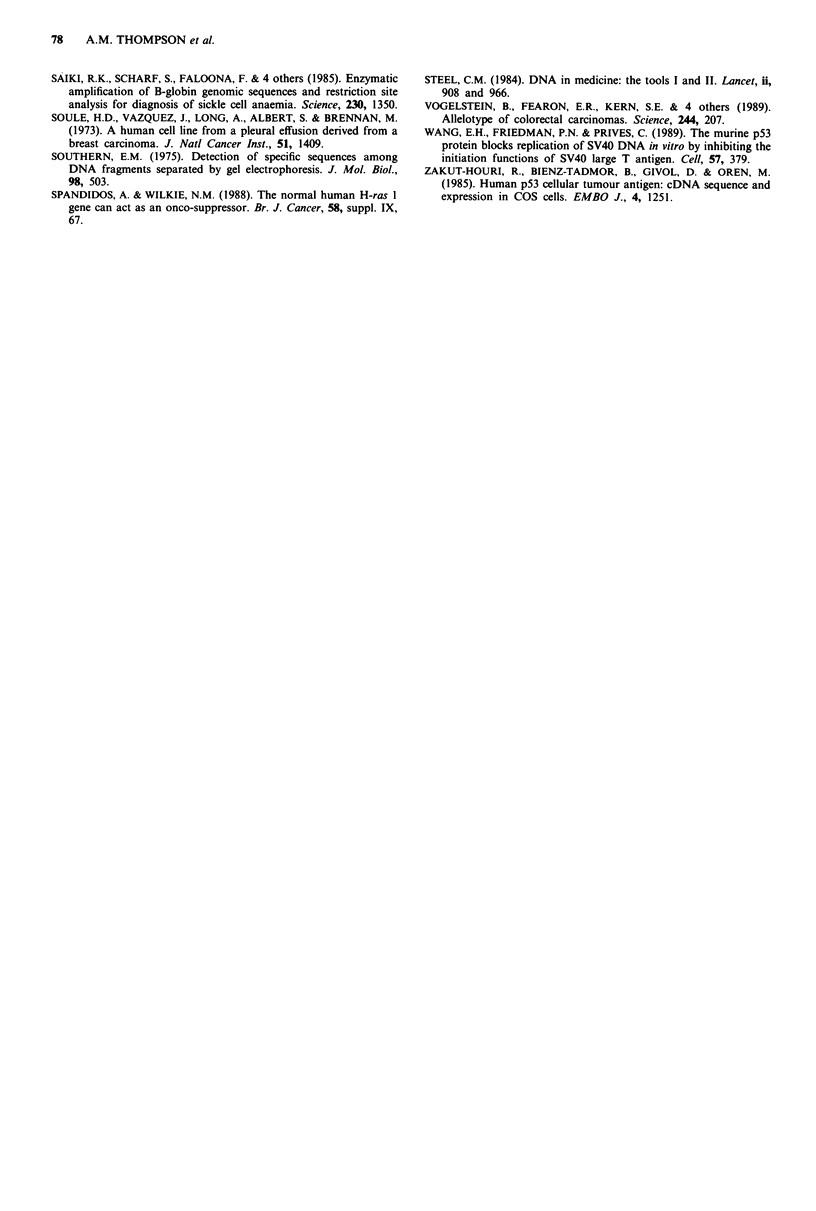# p53 gene mRNA expression and chromosome 17p allele loss in breast cancer.

**DOI:** 10.1038/bjc.1990.17

**Published:** 1990-01

**Authors:** A. M. Thompson, C. M. Steel, U. Chetty, R. A. Hawkins, W. R. Miller, D. C. Carter, A. P. Forrest, H. J. Evans

**Affiliations:** Department of Surgery, Royal Infirmary, Edinburgh, UK.

## Abstract

**Images:**


*Br. J. Cancer* (1990), **61**, 74–78 © Macmillan Press Ltd., 1990

### p53 gene mRNA expression and chromosome 17p allele loss in breast cancer

A.M. Thompson[1,2], C.M. Steel[2], U. Chetty[1], R.A. Hawkins[1], W.R. Miller[1], D.C. Carter[1], A.P.M. Forrest[3] & H.J. Evans[2]

[1]*Department of Surgery, Royal Infirmary, Edinburgh EH3 9YW;* [2]*MRC Human Genetics Unit, Western General Hospital, Crewe Road, Edinburgh EH4 2XU; and* [3]*Scottish Cancer Trials Office, University of Edinburgh, Medical School, Teviot Place, Edinburgh EH8 9JU, UK.*

**Summary** p53 messenger RNA expression was examined using a cDNA probe in 76 fresh primary breast tumour specimens, 15 of which came from patients treated with taxoxifen prior to surgery. A 2.8 kb mRNA for p53 was expressed in 43 of the 76 specimens. In 19 tumours the levels were similar to those seen in non-malignant (reduction mammoplasty) breast tissue, but in 24 tumours over-expression of mRNA for p53, approaching that seen in three breast cancer cell lines, was found. The cell lines MCF-7, T-47D and MDA-MB-231 expressed three p53 mRNA species of about 2.8 kb and a fourth of 1.6 kb. Increased mRNA expression for p53 correlated ($P < 0.05$) with loss of genetic material from the short arm of chromosome 17 as demonstrated by allele loss with the VNTR probe YNZ 22.1. There was also statistically significant correlation between increased p53 mRNA expression and low oestrogen receptor protein content in the tumours ($P < 0.05$), but not with other clinical parameters. The findings support the view that p53 is involved in breast tumour biology, and suggest that its role may be complex.

p53 is a 53 kDa phosphoprotein with a short half-life (5–45 min) (Reich & Levine, 1984; Halevy *et al.*, 1988). The role of p53 protein remains obscure although the p53 gene has the structural features of a 'housekeeping' gene, including absence of a TATA box (Reynolds *et al.*, 1984; Bienz-Tadmor *et al.*, 1985). p53 may modulate transcriptional activation by binding to DNA in a similar way to the *myc* protein (Donner *et al.*, 1982), allowing cells to progress from a growth arrested state to an actively dividing state or to bypass the need for platelet derived growth factor in the induction of competence (Oren, 1986).

Despite persuasive evidence for its role as an oncogene (Eliyahu *et al.*, 1984, 1985; Parada *et al.*, 1984; Editorial, 1988; Oren, 1986) there is also reason to believe that p53 can act as a tumour suppressor gene (Green, 1989; Wang *et al.*, 1989). This paradox may be resolved if rearrangements of the p53 DNA alter the structure, expression (Masuda *et al.*, 1987) or stability (Jenkins *et al.*, 1985) of the 53 kDa protein product and if the function of a mutated p53 product differs from that of the normal gene (Green, 1989). The p53 gene maps to the 13.1 region of the short arm of chromosome 17 (Miller *et al.*, 1986). One previous study (Masuda *et al.*, 1987) did not find detectable changes in the p53 gene in breast cancer. However, using the YNZ 22.1 probe (Nakamura *et al.*, 1988), which also maps near the tip of 17p at 13.3, Mackay *et al.* (1988) found most patients had two alleles in their constitutional (blood) DNA and 61% of these informative patients had demonstrable loss of one allele (loss of heterozygosity) in the tumour DNA.

p53 mRNA and p53 protein levels may (Reich *et al.*, 1983) or may not (Richon *et al.*, 1989) correspond, since regulation of p53 expression can occur at the level of mRNA abundance or of p53 protein stability, depending on the system under study (Rovinski *et al.*, 1987).

While previous work on human breast cancer has examined p53 protein expression (Cattoretti *et al.*, 1988) or chromosome 17p allelic loss (Mackay *et al.*, 1988; Devilee *et al.*, 1989), no previous study has attempted to link the two by relating expression of p53 mRNA to clinical parameters and/or to deletions of chromosome 17p in breast cancer.

Correspondence: A.M. Thompson, Department of Surgery, Royal Infirmary, Edinburgh EH3 9YW, UK.
Received 18 July 1989; and in revised form 5 September 1989.

### Materials and methods

Seventy-six patients with fully documented history, examination, staging investigations and follow-up, who presented with breast cancer to the University Department of Surgery Breast Unit at Longmore Hospital, Edinburgh, were studied. They comprised 61 untreated and 15 tamoxifen-treated consecutive breast cancer patients from whom sufficient material was available for analysis. Tumour tissues (minimum 0.2 g) from patients who underwent wedge biopsy, local excision or mastectomy for carcinoma of the breast were frozen in liquid nitrogen and stored at − 70°C. Tissue immediately adjacent to that stored was fixed for histopathology and a further piece of tumour submitted for oestrogen receptor assay. For comparison with constitutional DNA, 20 ml of venous blood was withdrawn for DNA extraction from white blood cells. Breast tissue from 10 patients who underwent cosmetic reduction mammoplasty and who did not have a personal or family history of breast cancer was also obtained fresh and immediately frozen.

The breast cancer cell lines MCF-7 (Soule *et al.*, 1973), MDA-MB-231 (Cailleau *et al.*, 1974) and T-47D (Keydar *et al.*, 1979) were cultured and maintained under mycoplasma-free (Barile, 1973) standard conditions. They were harvested in the logarithmic phase of growth and the RNA was extracted for comparison with that from the tumours.

### *Ribonucleic acid extraction*

From frozen tumour, total ribonucleic acid (RNA) was extracted using a modification of the method of Auffrey and Rougeon (1980). Briefly, a known weight of frozen tumour or a known number of cells washed in phosphate buffered saline was pulverised and then disrupted in 2 ml 100 mg$^{-1}$ 3 M lithium chloride, 6 M urea and precipitated at 4° C overnight. The DNA was sheared using a Soniprep 150 ultrasonic disintegrator (MSE Scientific Instruments, Crawley, UK) with an ice jacket, the RNA was recovered by centrifugation at 12,000 r.p.m. and the pellet was taken up in 6 ml of 10 mM Tris buffer pH 7.0, 0.1% sodium dodecyl sulphate (SDS). Three hundred μg of proteinase K (Boehringer Mannheim, FRG) was added and the sample was incubated at 37°C for 20 min. Protein was extracted using phenol equilibrated with Tris (0.1 M, pH 7) and chloroform:isoamylalcohol (24:1).

Following ethanol precipitation of the aqueous phase at − 20°C, the RNA was recovered by centrifugation and dissolved in autoclaved distilled water treated with diethyl pyrocarbonate (DEPC, Sigma, USA) and stored in aliquots at − 70°C. The quantity and purity of the RNA was assessed by spectrophotometry at 260 nm and 240 nm.

Throughout the RNA extraction procedures, sterile disposable plastic ware was used where possible; all solutions were made up with autoclaved DEPC-treated water using baked glassware and gloves were worn to minimise exogenous ribonuclease contamination (Maniatis *et al.*, 1982).

*Electrophoresis and transfer of RNA*

Twenty μg of total RNA was denatured with formamide and formaldehyde at 55°C for 20 min; 2 μl loading buffer (50% glycerol, 1 mM EDTA 0.4% bromophenol blue, 0.4% xylene cyanol) and 1 μl 10 μg $ul^{-1}$ ethidium bromide were added to each sample. The denatured specimens were loaded on to a 1.1% agarose gel containing 0.66 M formaldehyde, submerged beneath MOPS buffer (morpholinopropanesulphonic acid 0.2 M, pH 7.0, 50 mM sodium acetate pH 7.0, 5 mM EDTA) and the RNA species were separated electrophoretically (method modified from Fourney *et al.*, 1988). The gel was washed in two changes of 10 × standard saline-citrate (1 × SSC contains 150 mM sodium chloride, 300 mM sodium citrate, 1 mM EDTA, pH 7.4) and photographed under a UV transilluminator. The RNA was transferred to a nylon filter (hybond-N, Amersham, UK) by capillary action using 10 × SSC over 8 h (method modified from Southern, 1975). The filter was rinsed in 2 × SSC and air-dried, and the RNA was covalently fixed to the membrane using a UV transilluminator. The filter and remaining gel were photographed to check for adequate transfer of the RNA.

*Probe hybridisation*

Filters were pre-hybridised in 7% SDS, 0.5 M disodium hydrogen phosphate (pH 7.2) and 1 mM EDTA pH 7.0 (method modified from Church & Gilbert, 1984) for 30 min at 65°C. To this was added $^{32}$P-cytidine triphosphate (CTP) labelled cDNA probe, with specific activity to $10^7$ c.p.m. $ml^{-1}$ using a random prime DNA-labelling system (Boehringer Mannheim, FRG). $^{32}$P-CTP incorporated probe was separated from unincorporated radionucleotide using a Sephadex column (Nick column, Pharmacia, UK) and denatured before addition to the hybridisation solution.

To detect the p53 mRNA, the 2.1 kb cDNA clone php53Bam of p53 protein mRNA cut from pBR322 (Zakut-Houri *et al.*, 1985) was used. Following 24 h hybridisation, filters were washed to remove non-specifically attached probe in two changes of 0.1% SDS 10 mM disodium hydrogen phosphate wash buffer at 65°C with agitation. The filters were blotted dry, wrapped in clingfilm and exposed to pre-flashed Kodak XAR film at − 70°C for up to 14 days.

The extent of hybridisation of radiolabelled probe to the mRNA species was determined from densitometry (using a laser densitometer constructed by the Medical Research Council Human Genetics Unit) and expressed with respect to hybridisation to the actin probe. The size of each mRNA species was calculated from the position of ribosomal RNA markers.

The filters were stripped of residual probe by washing at 80°C for 60 min in 0.1% SDS and the filter was checked by autoradiography. As a standard probe, the Pst 1 insert cDNA of plasmid 91, detecting mouse a-actin mRNA-specific sequences (Minty *et al.*, 1981) was then hybridised and washed under the above conditions to quantify accurately the mRNA in each total RNA sample loaded.

*DNA extraction*

DNA was extracted from frozen tissue by disrupting finely chopped tissue in lysis buffer containing 1% SDS. Impurities were removed by using RNase and proteinase K, then phenol and chloroform, and the DNA was precipitated using ethanol in the presence of salt (Steel, 1984). Precipitated DNA was spooled from the alcohol, air-dried and redissolved in Tris/EDTA buffer, and the concentration and purity of the DNA were assessed using spectrophotometry at 260 nm and 280 nm. DNA was extracted from 20 ml venous blood in a similar way, but with an additional protein extraction and precipitation step prior to RNase treatment.

DNA (5 μg) from each patient's blood and tumour was digested using a bacterial endonuclease (for example, Tab I), the samples were separated electrophoretically alongside digested lambda markers on a 0.8% agarose gel, the DNA fragments transferred to a hybond-N membrane (Amersham, UK) using the Southern blot technique (Southern, 1975) and the DNA was fixed to the membrane with ultraviolet light and baking at 80°C for 2 h.

The membrane was incubated in hybridisation buffer (5 × Denhart's, 5 × SSC, 0.1% SDS, 10% dextran sulphate) to which $10^7$ c.p.m. $ml^{-1}$ $^{32}$P-CTP-labelled YNZ 22.1 insert was added (Nakamura *et al.*, 1988) and allowed to hybridise for 24 h.

Excess probe was washed from the membrane using successive washes of 0.1% SDS and 1 × SSC and the DNA fragments were detected by autoradiography at − 70°C to pre-flashed Kodak XAR film.

*Oestrogen receptors*

The oestrogen receptor content was measured using the Enzyme Immunoassay (EIA; kit from Abbott Laboratories, North Chicago, IL, USA) and expressed in fmol per mg protein for both the tumours and the cell lines. Oestrogen receptor protein concentrations of 20 fmol $mg^{-1}$ protein or greater were considered to be 'significant' (moderate to rich).

### Results

A 2.8 kb p53 mRNA was detected in 43 of the 76 tumour specimens (57%), in all three breast cancer cell lines and in six of the 10 reduction mammoplasty specimens (Figure 1). Low levels of this p53 mRNA (comparable to those found in the six positive mammoplasty specimens) were also detected in normal human tonsil, uterus and ovarian tissue (data not shown). There were quantitative differences between the tumour specimens and qualitative differences between the tumours and the cell lines. Thirty-three of the 76 patients had no detectable p53 mRNA in their tumour tissue and 19 patients had detectable p53 mRNA of 2.8 kb similar to quantity to the reduction mammoplasty specimens that gave a positive signal. Twenty-four patients had increased levels of the 2.8 kb p53 mRNA approaching those found in the cell lines.

**Figure 1** p53 mRNA expression in three breast cancer cell lines (MCF-7, MDA-MB-231 and T-47D), four representative breast tumours with no mRNA expression (0), normal mRNA expression (+) comparable to control (reduction mammoplasty) tissue and increased mRNA expression (+ + or + + +). In each case, upper part of plate shows p53 mRNA species and lower part of plate the control actin mRNA.

The three breast cancer cell lines each yielded four p53 mRNA species. Three closely related species were of approximately 2.8 kb, with differences between cell lines in the amounts of mRNA for each of these species. A fourth (1.6 kb) p53 mRNA was strongly expressed in all three lines.

There was a significant tendency for increased tumour p53 mRNA expression to be associated with clinically insignificant levels of oestrogen receptor protein ($P = 0.049$, $\chi^2$ test; Table I). No statistically significant correlation was found between p53 mRNA expression and tumour size, spread of the tumour to lymph nodes, histolopathological features of the tumour, patient age or menopausal status.

The 76 patients all yielded sufficient DNA for analysis from both venous blood and tumour. Using the php53Bam cDNA probe for p53, polymorphic bands were detected in less than 10% of samples with BamHI, Bgl II, Sca I, Ban II, HinIII, EcoRI or Taq I and no rearrangements were identified. However, with the YNZ 22.1 cDNA probe and Taq I digests, 52 of the 76 (69%) blood DNA samples were polymorphic and the remaining 24 were not informative. Among the 53 informative patients there was unequivocal loss of heterozygosity (loss or marked diminution in intensity of one allele) in 30 tumours (58%) when compared to the constitutive (blood) DNA (Figure 2).

Loss of genetic material from the tip of the short arm of chromosome 17, as determined by loss of heterozygosity using the YNZ 22.1 probe, was significantly correlated with increased p53 mRNA expression (Table II, $P = 0.04$, $\chi^2$ test).

Allelic loss was also correlated with low levels of oestrogen receptor protein ($P = 0.024$, Fisher's exact test). When the present data are combined with those from our earlier series (Mackay et al., 1988) the association becomes highly significant (Table III, $P < 0.01$).

**Table I** p53 mRNA expression compared to oestrogen receptor protein in 76 breast cancer specimens ($\chi^2$ 6.04, $P = 0.049$)

| *Oestrogen receptor* | *p53 mRNA expression* | | |
|---|---|---|---|
| *(fmol $mg^{-1}$ protein)* | *Nil* | *Normal* | *Increased* |
| Significant (>20) | 20 | 14 | 9 |
| Insignificant (<20) | 13 | 5 | 15 |

**Figure 2** Detection of DNA alleles using cDNA probe YNZ 22.1 following digestion of blood (B) tumour (T) DNA pairs with the endonuclease Taql, demonstrating no allelic loss (left pair) and loss of heterozygosity (right pair) from tumour DNA.

**Table II** p53 mRNA expression compared to allelic loss in 52 informative patients as demonstrated using the YNZ 22.1 probe for the short arm of chromosome 17 ($\chi^2$ 6.29, $P = 0.04$)

| | *p53 mRNA expression* | | | |
|---|---|---|---|---|
| | *Nil* | *Normal* | *Increased* | |
| Allele loss | 10 | 6 | 14 | (30) |
| No allele loss | 12 | 7 | 3 | (22) |

**Table III** Allelic loss in 52 informative patients with the YNZ 22.1 probe compared to oestrogen receptor protein ($P = 0.024$, Fisher's exact test)

| | *Oestrogen receptor (fmol $mg^{-1}$ protein)* | |
|---|---|---|
| | *Significant >20* | *Insignificant <20* |
| Allele loss | 11 (19) | 19 (32) |
| No allele loss | 15 (24) | 7 (11) |

Figures in parentheses include 34 informative patients from our previously reported data (Mackay et al., 1988; $P = 0.004$, Fisher's exact test).

### Discussion

This study has examined p53 mRNA expression and loss of genetic material from the short arm of chromosome 17 in 76 patients. A 2.8 kb mRNA for p53 was detected in 43 of the 76 breast cancer specimens. This corresponds to the mRNA for p53 identified in previous studies of human tissue (Harlow et al., 1985; Baker et al., 1989). The quantitative difference between tumours in p53 mRNA expression raises the possibility that the p53 gene may fulfil different functions in the patients with no detectable mRNA by comparison with those in whom there was normal or increased expression. In those tumours where no p53 mRNA was detected, this may reflect deficiency of normal (unmutated) p53 and hence a reduced tumour suppressor function. Deletion of one copy of the p53 gene is compatible with increased function of the other (abnormal) gene. Thus, where there is normal or increased p53 mRNA expression, this may be of a mutated form (for example a point mutation), which therefore acts as an oncogene, promoting carcinogenesis. Alternatively, loss of one allele may confer a minor growth advantage, with subsequent mutation of the remaining p53 required to initiate or promote carcinogenesis. If mutation were to occur first, loss of the normal allele may be required to allow effective expression of the mutant p53, by analogy with co-transfection studies of normal and mutated H-*ras* (Spandidos & Wilkie, 1988). The step between normal and mutated p53 cannot readily be established using the Northern blot technique, although future use of the polymerase chain reaction (Saiki et al., 1985) should clarify the situation.

The qualitative differences between the tumours and cell lines may reflect reading frame differences (likely to give rise to the 1.6 kb mRNA) or splicing, or even different adenylated tail lengths resulting in the three messages of about 2.8 kb in size. However, when MCF-7 cells are grown as xenografts in immunosuppressed mice, only a single 2.8 kb p53 mRNA species and no 1.6 kb mRNA is detected in the tumour tissue. This finding is independent of the rate of tumour growth (A.M. Thompson, manuscript in preparation). A single p53 mRNA species of 1.8 kb has been noted previously in NIH3T3 cells (Reich et al., 1983; Reich & Levine, 1984) and this mRNA may correspond to the nuclear mRNA regulating translation detected by Khochbin and Lawrence (1988).

The three cell lines examined are distinguishable on karyotype and on molecular analysis (using cDNA probes), and they have different phenotypic characteristics; for example, the MCF-7 line used in these studies has on average 120 fmol $mg^{-1}$ total protein oestrogen receptor protein, T-47D 40 fmol and MDA-MB-231 0 fmol. These lines also show differing sensitivity to oestrogens and anti-oestrogens. The consistently high level of p53 mRNA expressed in all three lines thus implies that, in this *in vitro* setting, p53 mRNA expression is independent of oestrogen receptor protein content of the cells and of hormone sensitivity. Although we have not confirmed the correlation noted *in vivo* between increased p53 mRNA expression and oestrogen poor tumours, this may be due to a myriad of factors including the divergence of cell lines from the original tumour with time.

Cattoretti *et al.* (1988), using an antibody PAb1801 specific for human p53 protein in breast cancer specimens, noted a correlation between oestrogen receptor negative tumours and elevated p53 protein expression ($P < 0.05$). Using this antibody, no other significant correlation was identified. These results therefore agree with our findings in relation to p53 mRNA.

This study confirms that the loss of one YNZ 22.1 allele from the short arm of chromosome 17 occurs in over half the breast tumours studied (Mackay *et al.*, 1988, Devilee *et al.*, 1989) and establishes for the first time that this allele loss correlates with clinically insignificant levels of oestrogen receptor protein. The tip of the short arm of chromosome 17 is thus of importance in breast as well as in colon cancer (Lothe *et al.*, 1988; Vogelstein *et al.*, 1989).

Although of all the highly informative probes available for this study, YNZ 22.1, located at 17p 13.3, was the closest to the p53 (17p 13.1) locus, it is still several megabases telomeric to the p53 gene. The correlation between loss of heterozygosity for YNZ 22.1 and p53 mRNA expression provides evidence that there may be some link between a putative gene conferring increased susceptibility to cancer (Mackay *et al.*, 1988) and the oncogene or tumour suppressor gene function of p53.

Eleven of the 76 patients had tumours showing loss of heterozygosity for YNZ 22.1, increased expression of p53 mRNA and low levels of oestrogen receptor protein. With just 12 months mean follow-up, two of these patients have already relapsed with metastatic disease. Continued follow-up of the whole cohort will establish the prognostic significance of the present findings.

The observation that, in almost a third of tumours, p53 mRNA levels were elevated while, in a comparable proportion, the message was not detectable, supports the view that p53 is involved in breast tumour biology. Whether this is as an oncogene or as a tumour suppressor gene (possibly as either, depending on the individual tumour) remains to be seen. The advent of highly polymorphic probes for the p53 gene and the application of more recent technology, such as the polymerase chain reaction, should resolve these issues.

The authors wish to thank Dr Y. Nakamura for probe YNZ 22.1, Dr M. Oren for probe php53Bam, Mrs I McKenzie, Mr C. Coles and Mrs P.A. Elder for technical assistance and Mr N. Davidson and colleagues for the photographic plates. A.M. Thompson was supported by the Scottish Hospitals Endowment Research Trust and a Faculty of Medicine Fellowship from the University of Edinburgh.

**References**

AUFFRAY, C. & ROUGEON, F. (1980). Purification of mouse immunoglobulin heavy chain messenger RNAs from total myeloma tumour RNA. *Eur. J. Biochem.*, **107**, 303.

BAKER, S.J., FEARON, E.R., NIGRO, J.M. & 9 others (1989). Chromosome 17 deletions and p53 gene mutations in colorectal carcinomas. *Science*, **244**, 217.

BARILE, M.F. (1973). Myoplasmal contamination of cell cultures. In *Contamination in Tissue Culture*, Fogh, J. (ed.) p. 140. Academic Press: New York.

BIENZ-TADMOR, B., ZAKUT-HOURI, R., LIBRESCO, S., GIVOL, D. & OREN, M. (1985). The 5′ region of the p53 gene: evolutionary conservation and evidence for a negative regulatory element. *EMBO J.*, **4**, 3209.

CAILLEAU, R., YOUNG, R., OLIVE, M. & REEVES, W.J. (1974). Breast tumour cell lines form pleural effusions. *J. Natl Cancer Inst.*, **53**, 661.

CATTORETTI, G., RILKE, F., ANDREOLA, S., D'AMATO, L. & DELIA, D. (1988). P53 expression in breast cancer. *Int. J. Cancer*, **41**, 178.

CHURCH, G.M. & GILBERT, W. (1984). Genomic sequencing. *Proc. Natl Acad. Sci., USA*, **81**, 1991.

DEVILEE, P., PEARSON, P.L. & CORNELISSE, C.J. (1989). Allele losses in breast cancer. *Lancet*, **i**, 154 (letter).

DONNER, P., GREISER-WILKE, I. & MOELLING, K. (1982). Nuclear localization and DNA binding of the transforming gene product of avian myelocytomatosis virus. *Nature*, **296**, 262.

EDITORIAL (1988). Genomic p53 gene immortalises. *Oncogene*, **2**, 419.

ELIYAHU, D., RAZ, A., GRUSS, P., GIVOL, D. & OREN, M. (1984). Participation of p53 cellular tumour antigen in transformation of normal embryonic cells. *Nature*, **312**, 646.

ELIYAHU, D., MICHALOVITZ, D. & OREN, M. (1985). Overproduction of p53 antigen makes established cells highly tumorigenic. *Nature*, **316**, 158.

FOURNEY, R.M., MIYAKOSHI, J., DAY, R.S. & PATERSON, M.C. (1988). Northern blotting: efficient staining and transfer. *Focus*, **10**, 5.

GREEN, M.R. (1989). When the products of oncogenes and anti-oncogenes meet. *Cell*, **56**, 1.

HALEVY, O., BEN-DAVID, A. & OREN, M. (1988). Analysis of p53 in lines derived from environmentally induced tumors. Oncogenes and Growth Control, 19–22 September 1988, European Molecular Biology Laboratory, Heidelberg, FR Germany.

HARLOW, E., WILLIAMSON, N.M., RALSTON, R., HELFMAN, D.M. & ADAMS, T.E. (1985). Molecular cloning and in vitro expression of a cDNA clone for human cellular tumour antigen p53. *Mol. Cell. Biol.*, **5**, 1601.

JENKINS, J.R., RUDGE, K., CHUMAKOV, P. & CURRIE, G.A. (1985). The cellular oncogene p53 can be activated by mutagenesis. *Nature*, **317**, 816.

KEYDAR, I., CHEN, L., KARBY, S. & 5 others. Establishment and characterization of a cell line of human breast carcinoma origin. *Eur. J. Cancer*, **15**, 659.

KHOCHBIN, S. & LAWRENCE, J.-J. (1988). Processing of p53 mRNA during induced differentiation of murine erythroleukaemia cells: is an altered splicing mechanism responsible for the post-transcriptional control of gene expression? *Gene*, **72**, 177.

LOTHE, R.A., NAKAMURA, Y., WOODWARD, S., GEDDE-DAHL, T. & WHITE, R. (1988). VNTR (variable number of tandem repeats) markers show loss of chromosome 17p sequences in human colorectal carcinomas. *Cytogenet. Cell Genet.*, **48**, 167.

MACKAY, J., ELDER, P.A., STEEL, C.M., FORREST, A.P.M. & EVANS, H.J. (1988). Allele loss on short arm of chromosome 17 in breast cancers. *Lancet*, **ii**, 1384.

MANIATIS, T., FRITSCH, E.F. & SAMBROOK, J. (1982). *Molecular Cloning: a Laboratory Manual*, p. 188. Cold Spring Harbor Laboratory: Cold Spring Harbor, NY.

MASUDA, H., MILLER, C., KOEFFLER, H.P., BATTIFORA, H. & CLINE, M.J. (1987). Rearrangement of the p53 gene in human oesteogenic sarcomas. *Proc. Natl Acad. Sci. USA*, **84**, 7716.

MILLER, C., MOHANDAS, T., WOLF, D., PROKOCIMER, M., ROTTER, V. & KOEFFLER, H.P. (1986). Human p53 gene localized to short arm of chromosome 17. *Nature*, **319**, 783.

MINTY, A.J., CARAVATTI, M., ROBERT, B. & 5 others (1981). Mouse actin messenger RNAs. *J. Biol. Chem.*, **256**, 1008.

NAKAMURA, Y., LATHROP, M., O'CONNELL, P. & 5 others (1988). A mapped set of DNA markers for human chromosome 17. *Genomics*, **2**, 302.

OREN, M. (1986). p53, molecular properties and biological activities. In *Oncogenes and Growth Control*, Kahn P. & Graf, T. (eds) p. 284. Springer-Verlag: Berlin.

PARADA, L.F., LAND, H., WEINBERG, R.A., WOLF, D. & ROTTER, V. (1984). Cooperation between gene encoding p53 antigen and *ras* in cellular transformation. *Nature*, **312**, 649.

REICH, N.C., OREN, M. & LEVINE, A.J. (1983). Two distinct mechanisms regulate the levels of a cellular tumour antigen, p53. *Mol. Cell. Biol.*, **3**, 2143.

REICH, N.C. & LEVINE, A.J. (1984). Growth regulation of a cellular tumour antigen, p53, in nontransformed cells. *Nature*, **308**, 199.

REYNOLDS, G.A., BASU, S.K., OSBORNE, T.F. & 5 others (1984). HMG CoA reductase: a negatively regulated gene with unusual promoter and 5′ untranslated regions. *Cell*, **38**, 275.

RICHON, V.M., RAMSAY, R.G., RIFKIND, R.A. & MARKS, P.A. (1989). Modulation of the c-*myb*, c-*myc* and p53 mRNA and protein levels during induced murine erythroleukaemia cell differentiation. *Oncogene*, **4**, 165.

ROVINSKI, B., MUNROE, D., PEACOCK, J., MOWAT, M., BERNSTEIN, A. & BENCHIMOL, S. (1987). Deletion of 5′ coding sequences of the cellular p53 gene in mouse erythroleukaemia: a novel mechanism of oncogene regulation. *Mol. Cell. Biol.*, **7**, 847.

SAIKI, R.K., SCHARF, S., FALOONA, F. & 4 others (1985). Enzymatic amplification of B-globin genomic sequences and restriction site analysis for diagnosis of sickle cell anaemia. *Science*, **230**, 1350.

SOULE, H.D., VAZQUEZ, J., LONG, A., ALBERT, S. & BRENNAN, M. (1973). A human cell line from a pleural effusion derived from a breast carcinoma. *J. Natl Cancer Inst.*, **51**, 1409.

SOUTHERN, E.M. (1975). Detection of specific sequences among DNA fragments separated by gel electrophoresis. *J. Mol. Biol.*, **98**, 503.

SPANDIDOS, A. & WILKIE, N.M. (1988). The normal human H-*ras* 1 gene can act as an onco-suppressor. *Br. J. Cancer*, **58**, suppl. IX, 67.

STEEL, C.M. (1984). DNA in medicine: the tools I and II. *Lancet*, **ii**, 908 and 966.

VOGELSTEIN, B., FEARON, E.R., KERN, S.E. & 4 others (1989). Allelotype of colorectal carcinomas. *Science*, **244**, 207.

WANG, E.H., FRIEDMAN, P.N. & PRIVES, C. (1989). The murine p53 protein blocks replication of SV40 DNA *in vitro* by inhibiting the initiation functions of SV40 large T antigen. *Cell*, **57**, 379.

ZAKUT-HOURI, R., BIENZ-TADMOR, B., GIVOL, D. & OREN, M. (1985). Human p53 cellular tumour antigen: cDNA sequence and expression in COS cells. *EMBO J.*, **4**, 1251.